# Use of strain ultrasound elastography versus fine-needle aspiration cytology for the differential diagnosis of thyroid nodules: a retrospective analysis

**DOI:** 10.6061/clinics/2020/e1594

**Published:** 2020-06-16

**Authors:** Xianghua Yang, Dongcai Zhai, Tao Zhang, Shenjie Zhang

**Affiliations:** Department of Doppler Ultrasonil, Xingtai People’s Hospital, Xingtai, Hebei, China, 054001

**Keywords:** Fine Needle Aspiration Cytology, Interobserver Variability, Strain Index, Thyroid Nodule, Ultrasound, Ultrasound Elastography

## Abstract

**OBJECTIVE:**

Fine-needle aspiration cytology is the risk stratification tool for thyroid nodules, and ultrasound elastography is not routinely used for the differential diagnosis of thyroid cancer. The current study aimed to compare the diagnostic parameters of ultrasound elastography and fine-needle aspiration cytology, using surgical pathology as the reference standard.

**METHODS:**

In total, 205 patients with abnormal thyroid function test results underwent ultrasound-guided fine-needle aspiration cytology on the basis of the American College of Radiology Thyroid Imaging-Reporting and Data System classification and strain ultrasound elastography according to the ASTERIA criteria. Histopathological examination of the surgical specimens was performed according to the 2017 World Health Organization classification system. Moreover, a beneficial score analysis for each modality was conducted.

**RESULTS:**

Of 265 nodules, 212 measured ≥1 cm. The strain index value increased from benign to malignant nodules, and the presence of autoimmune thyroid diseases did not affect the results (*p*>0.05 for all categories). The sensitivities of histopathological examination, ultrasound elastography, and fine-needle aspiration cytology for detection of nodules measuring ≥1 cm were 1, 1, and 0.97, respectively. The working area for detecting nodule(s) in a single image was similar between strain ultrasound elastography and fine-needle aspiration cytology for highly and moderately suspicious nodules. However, for mildly suspicious, unsuspicious, and benign nodules, the working area for detecting nodule(s) in a single image was higher in strain ultrasound elastography than in fine-needle aspiration cytology.

**CONCLUSION:**

Strain ultrasound elastography for highly and moderately suspicious nodules facilitated the detection of mildly suspicious, unsuspicious, and benign nodules.

## INTRODUCTION

Thyroid nodules are more common in the Chinese population, and patient evaluation must be performed to rule out malignancy (1). Only few individuals with thyroid nodules experience malignancy, and most of them present with papillary thyroid carcinoma (2). Therefore, benign thyroid nodules must be differentiated from malignant thyroid nodules.

Ultrasound features, such as irregular margins, hypoechogenicity, increased vascularization, regional lymphadenopathy, and microcalcifications, are associated with malignancy (3). However, when considered separately, none of these features have high specificity and sensitivity for the identification of thyroid nodules (2).

Fine-needle aspiration cytology is the risk stratification tool for thyroid nodules, and it is useful for the management of thyroid nodules (4). Moreover, it is a cost-effective and minimally invasive procedure that can be performed by an experienced professional (2). However, it yields a large number of inconclusive results (5).

Ultrasound elastography is a recently introduced technique. This procedure works on the principle of tissue elasticity assessment, and it can differentiate malignant (hard tissues) from benign (soft tissues) nodules (2). A prospective multicenter diagnostic study has reported that ultrasound elastography is effective for differentiating malignant from benign tumors (6). However, different degrees of fibrosis in the thyroid parenchyma and lymphocytic infiltration can influence tissue displacement during mechanical compression (7). Moreover, it is not routinely used in clinical practice.

This retrospective study of prospectively collected data aimed to compare the diagnostic parameters of strain ultrasound elastography *versus* fine-needle aspiration cytology for the differential diagnosis of thyroid nodules, with the use of surgical pathology as a reference standard.

## MATERIALS AND METHODS

### Ethics approval and consent to participant

The original protocol of the study (XPH/CL/15/19 dated September 4, 2019) was approved by the review board of Xingtai People’s Hospital. The study adheres to the guidelines of the Strengthening the Reporting of Observational Studies in Epidemiology for cross-sectional studies and the V2008 Declaration of Helsinki (Chinese version). All participants provided informed consent for diagnosis, radiological examination, biopsies, surgeries (if required), and publication of the study in all formats, which include personal data and images (if any) irrespective of time and language.

### Study population

**Figure 1:**
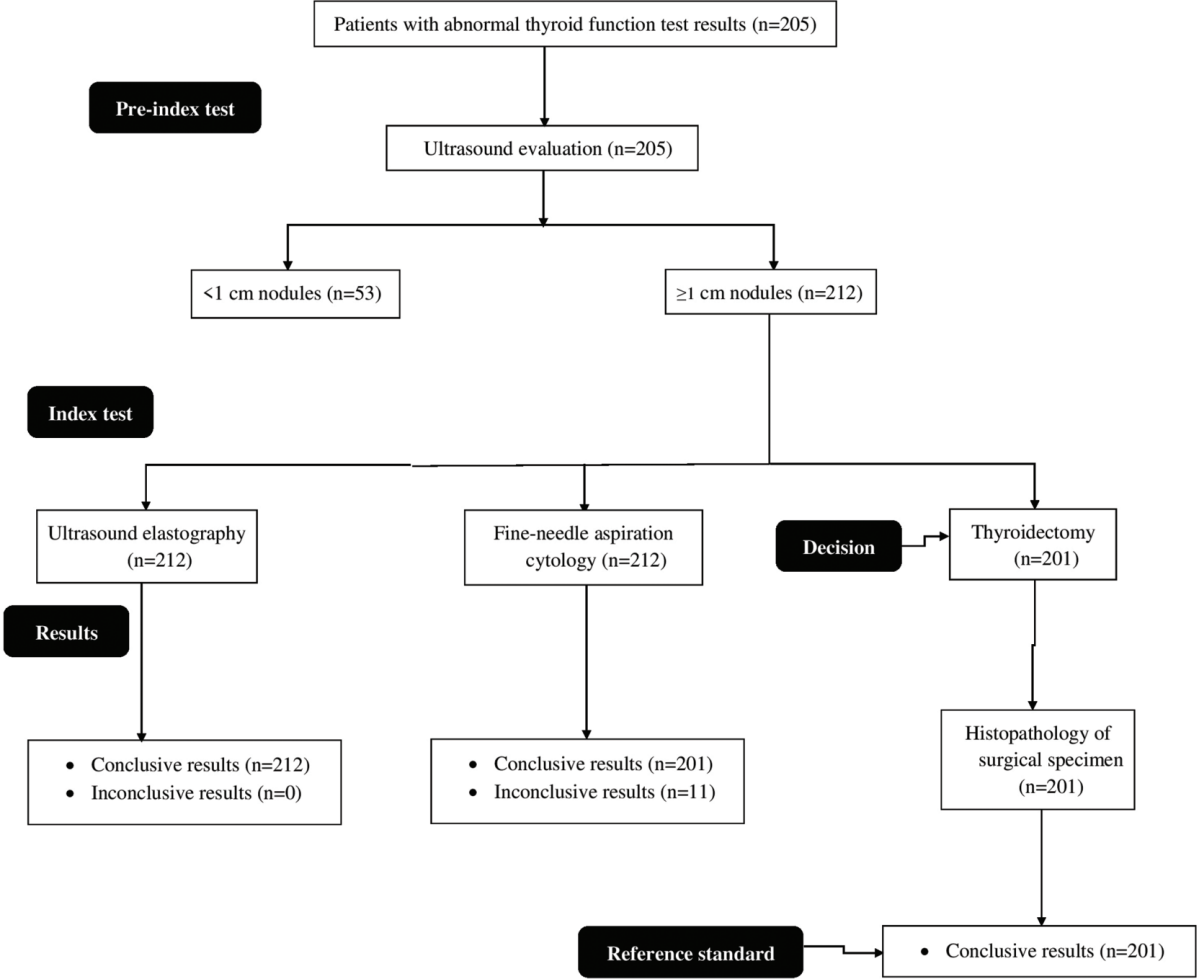
Flow diagram of the study.

From May 1, 2018, to July 30, 2019, 205 patients (aged 25-65 years) from the Department of Medicine of Xingtai People’s Hospital, China, and other referral hospitals were included in the study. The patients had available data on abnormal thyroid function test results (thyroid-stimulating hormone, free thyroxine, free triiodothyronine, calcitonin, anti-thyroglobulin antibody, anti-thyroperoxidase antibody, and anti-thyroid-stimulating hormone receptor antibody levels), and they presented with abnormal growth in the thyroid on the basis of neck examination. All patients underwent ultrasonography. In total, 178 patients presented with thyroid nodule(s) measuring ≥1 cm on the basis of the ultrasound examinations. Thereafter, the patients underwent ultrasound-guided fine-needle aspiration biopsies and strain ultrasound elastography. The flow diagram of the study is presented in Figure 1.

### Ultrasound evaluation

Thyroid ultrasonography was performed using a real-time ultrasound equipment (Resona 7, Shenzhen Mindray Bio-Medical Electronics Co., Ltd., Shenzhen, PR China) with a linear transducer (L11-3U, Shenzhen Mindray Bio-Medical Electronics Co., Ltd., Shenzhen, PR China) operating at 10-15 MHz. Ultrasonography was performed by ultrasound technologists, with a minimum experience of 5 years in thyroid imaging.

The nature (i.e., solid, cystic, and mixed type), echogenicity (e.g., isoechoic, hyperechoic, or hypoechoic with respect to the normal parenchyma of the neck muscles), homogeneity (homogeneous or inhomogeneous), size, microcalcifications (hyperechoic spots <2 mm without acoustic shadowing), and presence of an irregular margin and a halo sign (hypoechoic rim) of thyroid nodules were cautiously examined. The volume of the nodule was calculated using Equation 1 (2):



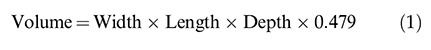



### Fine-needle aspiration biopsy

Under ultrasound guidance, biopsies were performed using 15-mm 25-gauge aspiration needles attached to a 5-mL syringe (DCHN-23-15.0, Cook Medical, Bloomington, IN, the USA). The solid mural of the nodule was collected based on suspicious calcification, hypoechogenic area, and/or presence of an irregular margin and halo sign (8). Biopsies were performed by endocrinologists with a minimum experience of 3 years.

### Strain ultrasound elastography

Strain ultrasound elastography was performed using the same ultrasound equipment and probe on the growth detected on the neck (whenever applicable). The probe was first placed on the neck in a transverse position, rather than a longitudinal position. Measurements in both positions were performed separately. In the area of interest, the probe was compressed (with light pressure) and relaxed two times per second. Then, it was moved 2-4 cm during compression and relaxation. Scores were assigned according to the ASTERIA criteria, as follows: 1: the area examined was homogenously green (elasticity in the whole area examined), 2: the area examined was light green and red with peripheral and central blue mass (the elasticity in the large portion of the examined area), 3: the examined area was blue with some light green and red mass (the large portion of the nodule with stiffness), and 4: the area examined was homogeneously blue (non-elastic nodule) (9). The color/score was considered if it was maintained for 15-20s on both positions and in four repetitions. The level of compression was kept constant throughout the examinations. The scores were as follows: 1: benign, 2: not suspicious, 3: mildly suspicious, 4: moderately suspicious, and 5: highly suspicious. The strain index (SI) was defined using Equation 2 (10). The average value of the three measurements in transverse and/or longitudinal views was considered for analyses. Ultrasound elastography was performed by ultrasound technologists. The size of the region of interest for measuring the strain index was standardized using the following equation:



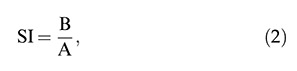



where B is the thyroid nodule strain and A is the strain of the softest area of the parenchyma.

The ultrasound indices were calculated and then used numerically using the device.

### Cytological examination

The aspirated material in ultrasound-guided fine-needle biopsies was air-dried and stained with May-Grunwald-Giemsa stain. Then, Based on the American College of Radiology Thyroid Imaging-Reporting and Data System, the results were classified as inconclusive (0), benign (1), unsuspicious (2), mildly suspicious (3), moderately suspicious (4), and highly suspicious (5) (11). The pathologists in our institutions were involved in the cytological examination. Patients with inconclusive or indeterminate specimens did not undergo repeat fine-needle aspiration biopsies.

### Thyroidectomy

All patients who had benign and suspicious results in ultrasound-guided fine-needle aspiration biopsies underwent partial or complete thyroidectomy under general anesthesia (3). The surgeons made an incision below the center of the neck. A part of or the whole thyroid gland with/without lymph nodes around was removed. Endocrine or head and neck surgeons with a minimum experience of 3 years performed the procedure. Patients with benign nodules also underwent surgery due to the lesion size and presence of symptoms.

### Histopathological examination of the surgical specimen

The surgical specimen was examined microscopically and was evaluated according to the 2017 World Health Organization classification for tumors of the endocrine organs (12). The pathologists were involved in the histopathological examination.

### Beneficial score analysis

The beneficial score analysis of each adopted modality was calculated using Equation 3 (13):



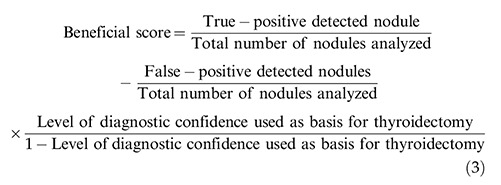



### Statistical analysis

InStat version Window 3.0.1 (GraphPad, San Diego, CA, the USA) was used for statistical analysis. The Fisher’s exact test was used to analyze categorical data (6). Fleiss kappa (k) statistic was used to determine interobserver variability, with consideration of the following k values: 0.1-0.2, slight agreement; 0.21-0.4, fair agreement; 0.41-0.6, moderate agreement; 0.61-0.8, substantial agreement; and 0.8-1, perfect agreement (14). A confidence interval of 95% was considered statistically significant.

## RESULTS

### Demographic and clinical characteristics of the participants

In total, 167 of 205 female patients had abnormal thyroid function test results. Moreover, 76 patients presented with autoimmune thyroid diseases, and only 55 (27%) had a family history of thyroid nodule(s). The other demographic and clinical characteristics of the participants are presented in Table 1.

### Ultrasound evaluation

In total, 265 nodules were analyzed via ultrasonography. Of them, 212 measured ≥1 cm. The ultrasound examination results of the nodules are presented in Table 2.

### Ultrasound elastography evaluation

**Figure 2:**
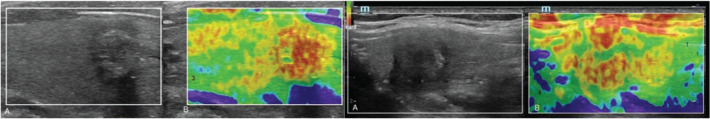
Strain ultrasound elastography. 1: The examined area was homogenously green (elasticity in the whole area examined; benign nodule), 2: the examined area was light green and red with peripheral and central blue mass (the elasticity in the large portion of the examined area; indeterminate follicular lesion), 3: the examined area was blue with some light green and red mass (the large portion of nodule with stiffness; nodule suspicious for malignancy), 4: the area was homogeneously blue (non-elastic nodule; malignant nodule). A: Real-time ultrasound evaluations. B: Ultrasound elastography evaluation. Real-time ultrasonography and ultrasound elastography were performed by ultrasound technologists with a minimum experience of 5 years.

The distribution of nodules on the basis of strain elastography ultrasound according to the ASTERIA criteria (Figure 2) is presented in Table 3.

**Figure 3:**
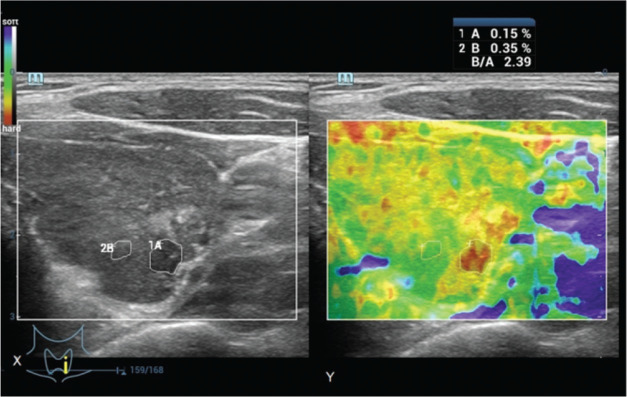
Determination of the strain index of a 42-year-old male patient with abnormal thyroid function test result. The B-to-A ratio was the strain index. X: Real-time ultrasonography. Y: Ultrasound elastography evaluation. B: Thyroid nodule strain. A: The strain of the softest area of the parenchyma. Real-time ultrasonography and ultrasound elastography were performed by ultrasound technologists with a minimum experience of 5 years.

**Figure 4:**
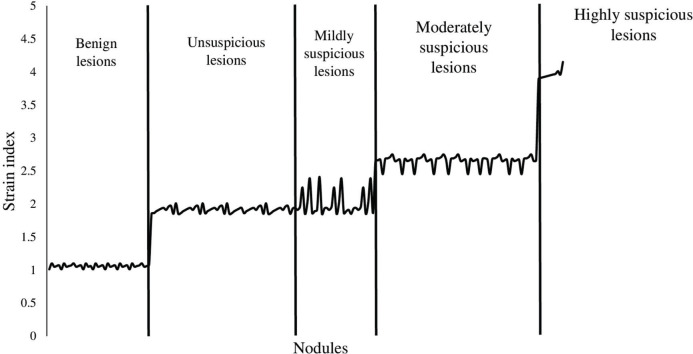
Distribution of the strain index according to the different categories of nodules. The ratio of thyroid nodule strain to the strain of the softest area of the parenchyma was considered the strain index. Ultrasound elastography was performed by ultrasound technologists with a minimum experience of 5 years.

The SI values for the nodules were 3.12±0.25 (Figure 3). The SI value was increased from benign to highly suspicious nodules. However, no significant difference was observed in terms of SI values between the unsuspicious and mildly suspicious nodules (Figure 4). The SI values for positivity and negativity to autoimmune thyroid diseases under the same categories were similar (*p*>0.05 for all categories).

### Fine-needle aspiration cytology

The distribution of nodules on the basis of fine-needle aspiration cytology is presented in Table 4.

### Histopathological examination of the surgical specimen

In total, 201 patients with nodules underwent surgery, and the histopathological results of the surgical specimen are presented in Table 5.

### Beneficial score analysis

Ultrasound elastography evaluation and histopathological examination of the surgical specimen had similar sensitivities. However, fine-needle aspiration cytology had a lower sensitivity than histopathological examination (1 *vs*. 0.97, *p*<0.0001, Table 6).

**Figure 5:**
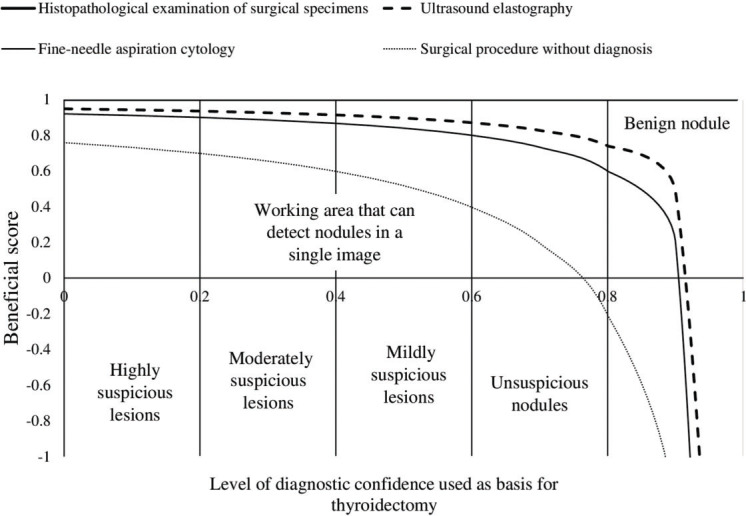
Beneficial score analysis. Pathologist with a minimum experience of 3 years performed the cytological and histopathological examinations. Ultrasound technologists with a minimum experience of 5 years in thyroid imaging conducted real-time ultrasonography and ultrasound elastography.

With the use of surgical pathology as a reference standard, the working area for detecting nodule(s) in a single image was similar between strain ultrasound elastography and fine-needle aspiration cytology for highly and moderately suspicious nodules. However, for mildly suspicious, unsuspicious nodules, and benign nodules, the working area for detecting nodule(s) in a single image was higher in strain ultrasound elastography than in fine-needle aspiration cytology. However, patients with benign nodules also underwent surgery, which was inappropriate (Figure 5).

### Interobserver variability

The interobserver variability for strain ultrasound elastography had a substantial agreement and that for conventional ultrasound examinations had a moderate agreement (Table 7).

## DISCUSSION

Strain ultrasound elastography and histopathological examination had similar sensitivities, and the presence of autoimmune thyroid diseases did not affect the SI values for nodules measuring ≥1 cm. The results of the current study were in accordance with those of the prospective studies (2,6,10) but not with those of the prospective study (15). Strain ultrasound elastography may be a good alternative to fine-needle aspiration cytology for the differential diagnosis of thyroid nodules.

The current study reported that the risk of malignancy increased with higher SI values, and this result was in accordance with that of prospective studies (2,10). Ultrasound-guided fine-needle aspiration cytology is generally performed for the differential diagnosis of thyroid nodules (8). However, the stiffness of thyroid nodules differs, and conventional ultrasound does not provide information about this characteristic (10). Strain ultrasound elastography can evaluate the stiffness of thyroid nodules (16). Thus, it may be an accurate method for the differential diagnosis of thyroid nodules.

With respect to the histopathological examination results, for mildly suspicious, unsuspicious, and benign nodules, strain ultrasound elastography had a comparatively high working area for detecting nodule(s) in a single image than fine-needle aspiration cytology. Ultrasound technologists may have less confidence in identifying suspicious margin, echogenic foci *versus* microcalcifications, and irregular margin in ultrasound images. In addition, fine-needle aspiration biopsies after cytopathology may have procedural issues (14). However, microcalcifications are found in both benign and suspicious nodules (16). Although strain ultrasound elastography may be less dependent based on the experience of operators (17), targeted education about sonographic findings may improve the interpretations of ultrasound images.

Moreover, strain ultrasound elastography, ultrasonography, and histopathological examination of the surgical specimen had substantial, moderate, and perfect agreement, respectively. The results of the current study were in accordance with those of retrospective analyses (14,17) and a prospective multicenter study (6). In the assessment of SI values, two regions of interest are required to maintain the same pressure (2), and strain elastography is pressure-dependent (16). Furthermore, it may be a reliable alternative for the differential diagnosis of thyroid nodules.

The current study had several limitations. That is, it is retrospective in nature, and a dynamic study was not performed. Moreover, all patients presented with nodules measuring >1 cm. However, thyroid papillary carcinoma can measure <1 cm. Only a small proportion of patients presented with malignant nodules. The cutoff SI values for the differential diagnosis of thyroid nodules were not evaluated, and these values are operator dependent (18). Finally, fine-needle aspiration cytology was performed only once in all patients.

## CONCLUSION

The conventional ultrasound could not differentiate benign from suspicious nodules. In addition, unlike that for conventional ultrasound and fine-needle aspiration cytology, the interobserver variability for strain ultrasound elastography showed substantial agreement. Strain ultrasound elastography for highly suspicious and moderately suspicious nodules facilitated the detection of mildly suspicious nodules, but not suspicious and benign nodules. Thus, it may be a more accurate and reliable alternative for the differential diagnosis of thyroid nodules than fine-needle aspiration cytology. However, a higher number of patients with calcified nodules are required to assess the hypothesis. Ultrasound elastography is a good auxiliary method to identify whether a nodule is a thyroid papillary carcinoma. Other types of malignant tumors, which may not be stiff, can develop in the thyroid. However, the significance of ultrasound elastography in identifying non-stiff malignant tumors is unclear.

## AUTHOR CONTRIBUTIONS

All authors read and approved the final version of the manuscript for publication. Yang X contributed to the formal analysis, resource acquisition, literature review, and manuscript drafting, review and edition for intellectual content. Zhai D was the project administrator and contributed to data validation and curation, supervision, and literature review. Zhang T contributed to the investigation, resource acquisition, software application and literature review. Zhang S contributed to the methodology design, supervision, data curation and literature review. All authors are accountable for all aspects of the work ensuring integrity and accuracy.

## Figures and Tables

**Table 1:** Demographic and clinical characteristics of the patients.

Parameters		Values
Medical records of the participants		205
Age (years)	Minimum	25
	Maximum	65
	Mean±SD	50.25±8.47
Gender	Male	38 (19)
	Female	167 (81)
*Serum thyroid-stimulating hormone level (mIU/L)		1.55±3.65
Family history	Yes	55 (27)
	No	150 (73)
**Serum free thyroxine level (ng/dL)		2.91±3.42
***Serum free triiodothyronine level (pg/dL)		675±85
****Serum calcitonin level (ng/dL)	Male	10.12±1.28
	Female	8.29±1.12
*****Serum anti-thyroglobulin antibody level (IU/mL)		35±12
******Serum anti-thyroperoxidase antibody level (IU/mL)		65±22
*******Serum anti-thyroid stimulating hormone receptor antibody level (IU/L)		3.12±0.34
#Autoimmune thyroid diseases	Positive	76 (37)
	Negative	129 (63)

Categorical variables are presented as frequency (percentage) and continuous variables as mean±SD.

* Normal value: 0.4-4 mIU/L.

** Normal value: 0.7-1.8 ng/dL for adults; 0.5-1 ng/dL for pregnant women.

*** Normal value: 260-480 pg/dL.

**** Normal value: <8.8 ng/dL for men; <5.8 ng/dL for women.

***** Normal value: <20 IU/mL.

****** Normal value: <35 IU/mL.

******* Normal value: <1.75 IU/L.

#At least two-times higher than the normal serum levels of anti-thyroglobulin antibody, anti-thyroperoxidase antibody, and/or anti-thyroid stimulating hormone receptor antibody.

**Table 2:** Ultrasound examination results of the nodules.

Parameters		Values
Medical records of the participants		205
Total number of nodules analyzed		265
Patients with <1 cm thyroid nodule(s)		27 (13)
Patients with ≥1 cm thyroid nodule(s)		178 (87)
Nodules measuring <1 cm in size		53 (20)
Nodules measuring ≥1 cm in size		212 (80)
Size (cm)	Minimum	0.61
	Maximum	5.82
	Mean±SD	2.32±0.58
Nature	Solid	163 (62)
	Cystic	41 (15)
	Mixed	61 (23)
Echogenicity	Isoechoic	47 (18)
	Hyperechoic	101 (38)
	*Hypoechoic	117 (44)
Homogeneity	Homogeneous	138 (52)
	Inhomogeneous	127 (48)
Microcalcifications		106 (40)
Irregular margin	Absent	43 (16)
	Present	222 (84)
Presence of a halo sign		53 (20)
Volume (cubic centimeter)	Minimum	0.75
	Maximum	42.51
	Mean±SD	9.51±1.21

Categorical variables are presented as frequency (percentage) and continuous variables as mean±SD.

* With respect to the normal parenchyma of the neck muscles.

**Table 3:** Distribution of nodules based on strain ultrasound elastography.

Differentiation	Values	
Total number of nodules subjected to ultrasound elastography	212	Strain index
Inconclusive	0 (0)	N/A
Benign	42 (20)	1.06±0.03
Unsuspicious	90 (42)	1.94±0.11
Mildly suspicious	66 (31)	2.63±0.13
Moderately suspicious	10 (5)	3.56±0.6
Highly suspicious	4 (2)	4.02±0.09

Categorical variables are presented as frequency (percentage) and continuous variables as mean±SD.

N/A: Not applicable.

Based on the ASTERIA criteria.

**Table 4:** Distribution of nodules based on fine-needle aspiration cytology.

Differentiation	Values
Total number of nodules subjected to fine-needle aspiration cytology	212
Inconclusive	11 (5)
Benign	35 (17)
Unsuspicious	90 (42)
Mildly suspicious	63 (30)
Moderately suspicious	9 (4)
Highly suspicious	4 (2)

Variables are presented as frequency (percentage).

According to the ACR TI-RADS classification.

Pathologist with a minimum experience of 3 years performed fine-needle aspiration cytology.

**Table 5:** Distribution of nodules according to the histopathological examination of the surgical specimen.

Differentiation	Values
Total number of nodules subjected to histopathological examination	201
Inconclusive	0 (0)
Benign	35 (17)
Unsuspicious	88 (44)
Mildly suspicious	64 (32)
Moderately suspicious	10 (5)
Highly suspicious	4 (2)

Variables are presented as frequency (percentage).

According to the 2017 WHO classification for tumors of the endocrine organs.

Pathologist with a minimum experience of 3 years conducted the histopathological examination.

N/A: Not applicable.

**Table 6:** Comparisons of diagnostic parameters.

Parameters	Histopathological examination of the surgical specimens	Ultrasound elastography		Fine-needle aspiration cytology	
Total number of nodules evaluated	201	212	* *p*-value	212	* *p*-value
True-positive detected nodules	201 (100)	201 (95)	0.0009	195 (92)	<0.0001
False-positive detected nodules	0 (0)	11 (5)		17 (8)	
Sensitivity	1	1	N/A	0.97**	<0.0001
Accuracy	1	0.948**	0.004	0.920**	0.0002

Variables are presented as frequency (percentage).

* With respect to the histopathological examination results of the surgical specimen.

N/A: Not applicable.

The Fisher’s exact test was used for statistical analysis.

A *p*-value <0.05 was considered significant.

Pathologists with a minimum experience of 3 years performed the histopathological examination.

Ultrasound technologists with a minimum experience of 5 years conducted ultrasonography.

** Significantly fewer than the histopathological examination of the surgical specimen.

**Table 7:** Comparisons of interobserver variability.

Kappa value	Ultrasound examinations	Fine-needle aspiration cytology	Ultrasound elastography	Histopathological examination of the surgical specimen
Observers	4	2	4	2
k	0.6	0.77	0.79	0.83

Pathologist with a minimum experience of 3 years performed biopsies and histopathological examination. Ultrasound technologists with a minimum experience of 5 years conducted ultrasound examinations.

k-value: 0.1-0.2, slight agreement; 0.21-0.4, fair agreement; 0.41-0.6, moderate agreement; 0.61-0.8, substantial agreement; and 0.8-1, perfect agreement.
